# Foregrounding Sociomaterial Practice in Our Understanding of Affordances: The Skilled Intentionality Framework

**DOI:** 10.3389/fpsyg.2016.01969

**Published:** 2017-01-09

**Authors:** Ludger van Dijk, Erik Rietveld

**Affiliations:** ^1^Amsterdam Medical Center, Department of Psychiatry and Amsterdam Brain and Cognition, University of AmsterdamAmsterdam, Netherlands; ^2^Department of Philosophy/Institute for Logic, Language and Computation, University of AmsterdamAmsterdam, Netherlands

**Keywords:** affordances, social coordination, ecological psychology, enaction, materiality, sociomateriality, Skilled Intentionality Framework, “higher” cognition

## Abstract

Social coordination and affordance perception always take part in concrete situations in real life. Nonetheless, the different fields of ecological psychology studying these phenomena do not seem to make this situated nature an object of study. To integrate both fields and extend the reach of the ecological approach, we introduce the Skilled Intentionality Framework that situates both social coordination and affordance perception within the human form of life and its rich landscape of affordances. We argue that in the human form of life the social and the material are intertwined and best understood as sociomateriality. Taking the form of life as our starting point foregrounds sociomateriality in each perspective we take on engaging with affordances. Using ethnographical examples we show how sociomateriality shows up from three different perspectives we take on affordances in a real-life situation. One perspective shows us a landscape of affordances that the sociomaterial environment offers. Zooming in on this landscape to the perspective of a local observer, we can focus on an individual coordinating with affordances offered by things and other people situated in this landscape. Finally, viewed from within this unfolding activity, we arrive at the person’s lived perspective: a field of relevant affordances solicits activity. The Skilled Intentionality Framework offers a way of integrating social coordination and affordance theory by drawing attention to these complementary perspectives. We end by showing a real-life example from the practice of architecture that suggests how this situated view that foregrounds sociomateriality can extend the scope of ecological psychology to forms of so-called “higher” cognition.

“… I distinguish between the movement of the waters on the river-bed and the shift of the bed itself; though there is not a sharp division of the one from the other.” – Wittgenstein (1969, §97)

## Introduction

In order to understand human social coordination in a Gibsonian framework it is important to understand in what sense affordances – possibilities for action provided to us by the environment (Gibson, 1979) – are always already situated in the sociomaterial practices that make up our human form of life; i.e., in what sense it is an affordance-in-sociomaterial-practice. Our approach is to combine such theoretical work on affordances with concrete real-life situations of social interaction as described in ethnography. As such this paper is targeted to anyone with an interest in affordances and skilled action, including skilled social action as we encounter that for example in ecological psychology, philosophy and various domains within the social sciences. Affordances, as “what [the environment] *offers* the animal, what it *provides* or *furnishes*, either for good or ill” (Gibson, 1979, p. 127), allow us to foreground sociomateriality because they do not occur in isolation. Rather, affordances are aspects of the ecological niche of a kind of animal. They thus always figure in a “setting of environmental features” (Gibson, 1979, p. 129), or of multiple affordances (p. 128).

Affordances are thus always situated. Looking around one will notice things and people offering multiple possibilities for action. Siting in the train for example, I can notice the possibility to drink from a bottle of water, talk to a fellow traveler or return to writing this paragraph. All these affordances belong to a wider socio-cultural context (Hodges and Baron, 1992; Costall, 1995; Ingold, 2000; Heft, 2001; Rietveld, 2008a). For example, I am seated in a “silence area,” so talking is not really an option and the bottle of water is not mine, but belongs to my neighbor, so I better not drink from it. Doing not much else than writing in this silence area, I am thus showing a responsiveness to a whole socially significant situation – to the kind of place in which I am situated, to a “behavior setting” (Heft, 2007, 2011). Moreover by typing away in appropriate silence, I contribute to maintaining this behavior setting as a “silence area” indeed. This way of responding and contributing to the maintenance of a behavior setting is a form of social coordination, albeit different from what is typically studied in ecological psychology, because it acknowledges social aspects even in situations where there is no direct interacting with other individuals (but see Barker, 1968; Heft, 2001).

More orthodox forms of social coordination are also found in our example and these are situated as well. In the behavior setting that is the train’s silence area, I am actually continuously adjusting my feet and leg placement to accommodate the person opposite me. I pretend not to be annoyed when someone’s phone rings, and exchange a brief glance of understanding with someone else equally annoyed. Adapting continuously to the people around me, I let the situation constrain my behavior, and by doing so (still typing in appropriate silence) I again do my part to maintain it. In fact, for any person in this silence area, being responsive to the behavior setting includes both being responsive to the opportunities for action, the affordances, offered by the material environment and to the opportunities for social engagement offered by other people. As we will show, there is no clear separation of the two because in acting skillfully one is attuned to the situation as a whole.

Despite the fact that in such real-life situations affordances and social coordination are situationally intertwined, the contextually situated nature of both is easily overlooked. An affordance for e.g., stair climbing (Warren, 1984) would be responded to very differently if one hopes to get up the old squeaking stairs without waking anyone, and the way one approaches and stops for a red light in a car (Fajen, 2007) changes if one is driving with one’s elderly mother-in-law or with a newborn baby in the backseat (see Hodges, 2007). Similarly, judging whether something can be carried together (Richardson et al., 2007) might change as soon as such judgment is required in a different context – say for carrying a coffin at a funeral. The dynamics of the coordinating people that actually carry a coffin would no doubt change as well. In other words, to understand how we respond to affordances offered both by material aspects of the environment and by other people, it is crucial that we understand the practical situation in which such behavior occurs.

Within ecological psychology there is currently a divide between the field of research that focuses on engagement with the affordances offered by the material environment and the field that focuses on social coordination. Moreover, as paradigmatic cases and dedicated methods are developing largely independently in both areas of ecological psychology, they risk focusing on a limited set of phenomena and growing apart further. The above examples, however, indicate that neither work that focuses on affordances in isolation nor on social coordination on its own, can account for the full breadth of skilled involvement of humans in the context of their ecological niche.

In fact, both fields are already focusing mostly on cases of direct “online” behavior (responding either on a current affordance or to another person), but neither foregrounds the larger situational context in which these dynamics unfold. Because of that, ecological psychology has yet to move into the domain labeled “oﬄine” or “higher” cognition, such as dealing with non-existent things (i.e., “representation hungry” problems – see Clark and Toribio, 1994; see also Rietveld and Kiverstein, 2014; Rietveld and Brouwers, 2016; Van Dijk and Withagen, 2016). To get the most out of ecological theory and extend ecological psychology beyond its current scope, we believe we need a framework that integrates both fields in a fundamental way. Doing so, we will claim, requires an understanding of individuals as coordinating with and situated in multiple nested scales of sociomaterial dynamics. We need to understand the human eco-niche as being *sociomaterial* through and through. Having such an integrative account, we believe, will not only bring the two parts of ecological theory closer together, but will also allow ecological theory as a whole to broaden its scope to include the wide variety of human practices.

To provide such an integrative account and broaden the scope of ecological psychology we will introduce the Skilled Intentionality Framework (SIF). Skilled Intentionality is coordinating with multiple affordances simultaneously. Our main point will be to show that the SIF integrates social coordination and affordances in a fundamental way because it incorporates the notion of sociomateriality. We will do so by first explaining the concept of sociomateriality as found in the field of ethnography in Section “Sociomaterial Entanglement.” After this preliminary, we will introduce three complementary perspectives on the human practices and the affordances they imply. In Section “Practices and the Landscape of Affordances” (i) a zoomed out perspective on our human practices as a whole will be provided in which the materiality and standing practices can be identified that constrain an individual’s activities. Complementing this perspective the section goes on to discuss (ii) a zoomed in perspective of a local observer looking at people acting in their environment. This second perspective highlights how individuals coordinating together continuously restructure sociomateriality. In Section “Skilled Intentionality” (iii) a third perspective is added: the *lived perspective* of a skilled individual being responsive to its surroundings.

Each of these perspectives on the human form of life brings an aspect of human involvement into view. On its own, however, any one perspective also loses sight of other aspects of situated coordination, which is the reason why we believe they are best treated as complementary perspectives. The metaphor of zooming in and out enables us to see how the SIF combines these perspectives to arrive at a rich framework for understanding a wide range of human involvement in ecological terms. Using an ethnographical example of the sociomaterial engagement of architects in practice, we will end Section “Skilled Intentionality” by illustrating how integrating social coordination with affordance-theory in a fundamental way, can open up ecological theory to dealing with “hard cases” of the *“whole realm of social significance*” as Gibson (1979, p. 128, our italics) called it. In the case of architecture this includes real life situations of dealing with non-existent things, such as a vision, drawing or model for a future building.

## Sociomaterial Entanglement

The neighbor’s bottle of water encountered while riding the train offered the opportunity to drink from, even though it would have been highly inappropriate to do so. It offered this affordance rather than, say, throwing it out of the window. The affordance of drinking from a bottle has, as Costall (1997) called a “canonical” character. Crucially, this canonical character comes from the bottle figuring in a large “constellation” of practices as Costall (2012, p. 91) calls it, shared among many individuals. In this way canonical affordances:

“characterize the normative character of the meaning of things. A chair, for example, is for sitting on, even though it may be used in many other ways, e.g., as firewood or for standing upon. The meaning of a chair is defined by its name, sustained and revealed within certain practices, and realized in its very construction.” (Costall, 1997, p. 97)

What Costall’s notion of canonical affordances stresses is the fact that such affordances are situated not just in the “current” behavior setting, but also in a more encompassing, shared and historically developed constellation – such affordances exist as they persist in shared and social *practice*s (see also Ingold, 2000, pp. 167–168). They exist as many individuals act on them in more or less appropriate ways, in the totality of practices that, together with other affordances, sustain them. For example, citing Dreyfus (1988), Costall points out that a hammer will only be perceived as such against the background of dealing with nails, walls and, say, pictures that afford hanging; i.e., against, what Heidegger (1927/1962, p. 97) called, a “totality of equipment.” As such, canonical affordances are part of what we might call a wider “standing practice”; they are relatively persistent material aspects of the practices in our shared socio-cultural environment, depending on an entire community of people, yet on no individual in particular.

### Sociomateriality in Practice

In the view that we are developing in this paper, relevant aspects of the environment and of the organism can only be understood in a concrete situation within a constellation of practices. Acknowledging these practices allows us to place socio-cultural aspects of our coordination center stage. However, material aspects of the environment equally partake in the constellation of practices. To see this, the taken for granted conception of *materiality* as “pre-formed substances” (Orlikowski, 2007, p. 1438) needs to be reconsidered.

Consider for instance research in the social sciences concerned with workplace practices. This research emphasizes how social practices are “inherently bound-up with materiality” such as places, material artifacts, bodies, and, infrastructures (Orlikowski, 2007, p. 1436). Zooming in on concrete situations by means of ethnography, the material and the social turn out to be intertwined in ways that lead researchers undertaking ethnographic studies to speak of “sociomaterial practices” (Mol, 2002; Suchman, 2007). Ethnographer Annemarie Mol illustrates this intertwinement well in discussing her ethnographic work in medical practice:

“[T]he practice of diagnosing and treating diseases inevitably requires cooperation. … In the consulting room something is *done*. …[T]wo people are required. A doctor and a patient. … The doctor must ask questions and the patient be willing and able to attend to answer them. And in addition to these two people there are other elements that play a more or less important role. The desk, the chairs, the general practitioner, the letter: they all participate in the events … As does [the patient’s] dog, without whom she might not have even tried to walk more than the fifty meters after which her left leg starts to hurt.” (Mol, 2002, pp. 22–23).

This example shows that the particular details (form the desk and the chairs to the patient’s dog) of the situations in which we act, matter a lot, which is why the constellation of practices shows up as sociomaterial in nature. It is only within the context of this situation that one of the people is primarily a “patient” (rather than say a grandmother or a love interest) and that the piece of paper shows up as an important “letter” from the general practitioner rather than as offering the opportunity to fold it into an airplane. More generally, ethnographic research like this shows that:

“all practices are always and everywhere sociomaterial, and … this sociomateriality is constitutive, shaping the contours and possibilities of everyday organizing.” (Orlikowski, 2007, p. 1444)

The “possibilities of everyday organizing,” the *affordances* encountered in daily life in the human ecological niche, form within our practices and these affordances are therefore always and everywhere sociomaterial. For example, we saw already that hammers are not just heavy things – their canonical character emphasized that they afford hammering relative to a socio-cultural practice. Moreover now we can see how this in turn affects its materiality: hammers are not just heavy, hammers are heavy *enough*, they have the *appropriate* weight to drive in a nail – a hammer’s materiality is *socio*materiality (see also Heidegger, 1927/1962, p. 98). In the context of our practices, the aspect of the sociomaterial environment also known as a “hammer” affords driving in nails. To be skillful in dealing with a hammer then, is to know your way about a particular aspect of the human *form of life* (see Wittgenstein, 1953, §123; see also Rietveld and Kiverstein, 2014; Van Dijk and Withagen, 2014).^1^

### Constitutive Entanglement

According to the social scientists quoted above, possibilities for everyday action are sociomaterial in a *constitutive* way, that is:

“A position of constitutive entanglement does not privilege either humans or technology (in one-way interactions), nor does it link them through a form of mutual reciprocation (in two-way interactions). Instead, the social and the material are considered to be inextricably related — there is no social that is not also material, and no material that is not also social.” (Orlikowski, 2007, p. 1437)

This is of fundamental importance and requires some unpacking. The notion of a constitutive entanglement is characterized by two features. First, its various aspects are interdependent and second, it emphasizes that none of the aspects has *priority* (is “privileged”) over another (see also Ter Hark, 1990; Schatzki, 1996; Mol, 2002, p. 133; Orlikowski, 2007; Ingold, 2011; Van Dijk, 2016; Van Dijk and Withagen, 2016). The ethnographic examples above that stress how the social implies materiality and materiality implies the social offer a case in point. But let us look at both features more closely by considering the relations between practices, affordances, activities and sociomateriality.

The relation between a practice and the affordances implied by it can be understood constitutionally. It is an example of a constitutive relation because (i) the practice and the affordances that take shape within it are interdependent: any affordance will imply a practice for realizing it and any practice will imply a landscape of available affordances.^2^ Furthermore (ii) practices and affordances do not admit of a prioritization. As we saw above, the affordance to use a hammer is available within the context of our hammering practices and conversely, the hammering practices are maintained by responsiveness to the possibilities for driving nails into walls.

The relation between parts and whole, we encounter here in the form of activities and practices respectively, can also be understood in constitutive terms. Consider for example the activities and the individuals partaking in a practice – for example the medical practice we saw in the ethnographic example above. As the situation in the doctor’s office unfolds, some person is primarily a “patient,” papers become important “letters,” reciprocally, in doing so, in *acting*, the behavior setting at the doctor’s office is maintained: thus when someone walks in unexpectedly for example, he will immediately adjust his behavior to fit in with the reserved atmosphere. The medical practices are thus maintained by the activities of the individuals within it. What the constitutive reading stresses is that the whole (the practices) gives form to its parts (the activities unfolding within it) and these activities equally give form to the practices as a whole. In general, in a constitutive entanglement the parts are continuous characteristics of a process – this process then is the continuously forming whole^3^ (see Barker, 1968; Shotter, 1983; Ingold, 2000, 2011; Van Dijk, 2016).

What this constitutive entanglement highlights, and ethnographic research helps to make tangible, is that we can take multiple complementary perspectives on the constellation of practices and that we can foreground the sociomateriality in each. First, the fact that in this view practices and affordances are two sides of the same coin, i.e., of the same sociomaterial entanglement of people, activities, places and things, allows us to switch between foregrounding the one or the other. Second, the idea that (individual or joint) activities are constitutionally related to (communal) practices allows us to conceptualize their differences as one of degree rather than kind. As researchers, we can thus think of ourselves as zooming in and out in both space and time on a form of life, to bring different aspects of it into prominence: practices or activities, affordances or their sociomateriality.

To unpack this view further and to bring its implications to bear on the relation between social coordination and affordances in ecological psychology, we will now introduce the Skilled Intentionality Framework that has sociomateriality at its heart. Through the SIF we will show how we can take (i) the zoomed out perspective on (relatively) persistent sociomateriality of a whole form of life (see The Zoomed Out Perspective: Practices and Affordances), (ii) a zoomed in perspective of a local observer looking at sociomateriality in flux (see Zooming in on the Landscape of Affordances) and (iii) the perspective from within an unfolding action as an individual responds to the multiple affordances available to him or her (see The Unfolding Action from the Actor’s Lived Perspective). Bringing these perspectives together, we will show in Section “Skilled Intentionality Unfolding in Architectural Practice” how they integrate social coordination and affordances such that ecological psychology will be open to account for real life situations of dealing with non-existent things, such as modeling a future building.

## Practices and the Landscape of Affordances

Wittgenstein’s concept of the ‘form of life’ fits in nicely with the constitutional entanglement and the constellation of practices as we identified them in ethnography and Costall’s work. The form of life of a kind of animal, as Rietveld and Kiverstein (2014, p. 328) point out, “consists of patterns in its behavior, i.e., relatively stable and regular ways of doing things.” Such relatively stable ways of doing things show themselves for example in the regularities that characterizes expert practices like architecture, surgery and academia as well as in our everyday activities, such as our appropriate use of chairs or doors and the way we talk about them (Wittgenstein, 1969, §7).

The constitutive character of the relation between activities and the standing practices, i.e., the form of life, implies that *activities* are sensible aspects only *relative to the form of life*. An example of this would be our human form of life in which we use, e.g., chairs for sitting and doors to enter or close off a room. Chairs do not play a role in the forms of life of, say, lions or earthworms, but they are relevant in our form of life. Indeed, were we to show genuine surprise or disbelief each time we encountered a chair, we would act *inappropriately* in a strong sense: we would fail to make sense because we fail to share with others a way of acting, of responding to, everyday things – that is, we fail to share agreement in our form of life, which is an agreement in what people typically *do* (Wittgenstein, 1953, §123; see Ter Hark, 1990, p. 70; Schatzki, 2002). The meaning and relevance of our activities are constrained by the form of life in which they figure.

To Wittgenstein, through our concrete activities of talking and doing (Moyal-Sharrock, 2004) both in everyday life and in expertise, a *river-bed of practices* continuously shows itself. These practices constrain activities – talking and doing – that unfold within it (Rietveld, 2008a), just as the movements of the water is constrained by the river-bed. Reciprocally, the movement also allows for shifts in the river-bed itself:

“… I distinguish between the movement of the waters on the river-bed and the shift of the bed itself; though there is not a sharp division of the one from the other” (Wittgenstein, 1969, §97)

The Skilled Intentionality Framework (Rietveld, 2012; Bruineberg and Rietveld, 2014, Figure 1; Rietveld and Kiverstein, 2014; Kiverstein and Rietveld, 2015; Rietveld et al., 2016) aligns with Wittgenstein and identifies the constellation of sociomaterial practices we encountered above as our human *form of life*. The form of life consists, in other words, of our actively maintained standing practices – our regular ways of doing things:

“What is common to human beings is not just the biology we share but also our being embedded in sociocultural practices: our sharing steady ways of living with others, our relatively stable ways of going on.” (Rietveld and Kiverstein, 2014, p. 329)

By taking up the Wittgensteinian concept of the *form of life*, the SIF opens up ecological psychology to sociomaterial aspects of the world. It includes the constraining influence of material properties but also our shared practical understanding of the affordances offered by buildings, chairs, silence areas and other people (Rietveld and Kiverstein, 2014, p. 329 ff.; see Kiverstein and Rietveld, 2015, p. 15).

### The Zoomed Out Perspective: Practices and Affordances

Notice that the form of life, as relatively stable and regular patterns of behavior, is perfectly concrete. We can see this clearly when we as behavioral scientists or philosophers “zoom out” from an individual’s activity in a sociomaterial situation to a perspective that allows us to discern patterns in the behavior of a community of people of a larger spatiotemporal grain. Think, for example, of the regularities one would notice when watching a time-lapse recording from above on New York’s Paley park or Amsterdam’s Vondelpark (see Whyte, 1980; Rietveld and Kiverstein, 2014). Regular ways of doing things appear across, e.g., seasons or times of day, and depend on, for example, the sociomaterial aspects of the environment such as benches, ponds and paths. For example, we will see people walk on paths and to a lesser extent on grass, but not on ponds, except when the water of the ponds is frozen.

We will call this view that aims to overlook such regularities of the form of life from a time-lapse camera a “zoomed out” perspective. Zooming in we see individuals caught up with people and things in multiple ongoing activities, but zooming out we notice their regularities: persistent practices – that is to say, the stable patterns of behaving that characterize the form of life. By calling attention to the form of life, the SIF aims to make the regularities that our activities exhibit tangible, and show how *these regularities are sociomaterial and therefore aspects of the environment that are available for coordinating with*.

Now in order to have a notion of affordances that acknowledges these large scale regularities and that is therefore open to sociomaterial practice, affordances are defined within the SIF as related to the form of life: an affordance is a relation between an aspect of the sociomaterial environment in flux and an ability available in a form of life (Rietveld and Kiverstein, 2014, p. 335; see also cf. Chemero, 2009; Rietveld, 2016; Rietveld and Brouwers, 2016). From this definition it is clear that this conception of affordances aims to emphasize the entanglement of the ever changing sociomaterial environment and the abilities that continuously form within this environment. Note that the definition does not imply a prior separation of its relata.

Defining affordances relative to a form of life turns the materiality of affordances into sociomateriality in the human case. It allows us to make sense of a chair not just as a place to sit but, as we shall see, as a *chair* as it figures in its many ways in our human practices, inviting sitting, but also naming, pointing to or marveling at in a museum (Withagen et al., 2012; Rietveld and Kiverstein, 2014). Similarly, doors are not only hinging vertical surfaces, but are *doors* that can solicit opening or keeping closed. And, as we shall see, it allows us to understand how stones can afford throwing in one situation and afford being a paper weight in the next. All these affordances are situated, but concretely available aspects of the sociomaterial environment to coordinate to. We can see that they function in these manifold ways if we zoom out on our practices in space and time to notice how chairs, doors, benches, paths and ponds are entangled within and across concrete situations.

Skilled Intentionality Frameworks sociomaterial notion of affordances is a more inclusive notion of affordances than the traditional purely “material” one. Nonetheless, these affordances still pose enormous (material and social) constrains on the possibilities available to an individual, to the extent that their materiality can appear to be “subject to no alteration or only to an imperceptible one” (Wittgenstein, 1969, §99). Thus although an office chair not only allows sitting, but now also affords for example calling it “an office chair,” the material reality of this aspect of the socio*material* environment still does not allow us to fly to Baghdad on it (cf. Cutting, 1982). So the SIF’s notion of affordances is constrained by material reality. As we will explain below and in the next section, however, the situated nature of encountering a chair as sociomaterial allows us to also make sense of the fact that a skilled individual will typically be constrained even further – he or she will for example not be inclined to point and call out “an office chair!” save when surprised to find one, as for example in a museum (compare this to a young child). In short, the SIF considers our human actions to be constrained by (and responsive to) not just the environment’s materiality, but its (broader and irreducible) sociomateriality.

### Zooming in on the Landscape of Affordances

Having defined affordances relationally and in terms of the form of life, an important re-orientation realized by the Skilled Intentionality Framework is that it allows us to switch between the standing *practices* and the *affordances* that they imply. Given that in our human form of life there are many aspects of the sociomaterial environment and many abilities available, these standing *practices* can thus be seen as unfolding in a relatively persisting rich “landscape of affordances” (Rietveld and Kiverstein, 2014).

By calling attention to the landscape of affordances within a form of life, the SIF allows us not only to understand the practices from the perspective of the affordances that they imply (in the park, for example, including the action possibilities offered by benches, paths and ponds), but it also allows us to zoom in on concrete and situated *activities* that constitute the various grains of the form of life (see Rietveld and Kiverstein, 2014). Specifically, we can zoom in to the spatiotemporal grain where individuals live (e.g., sitting on benches but not swimming on benches) – that is, the *zoomed in perspective* of the local observer. This is the perspective where, e.g., the dynamics of a behavior setting, a place or another concrete situation unfold as observed from the perspective of a behavioral scientist or as modeled by coordination dynamics.

Notice, however, that this zoomed in perspective, while highlighting the unfolding dynamics, obscures large-scale regularities. Just as one cannot observe someone’s habits if one just observe the person for a few seconds, one cannot observe a *practice* by watching it briefly. From the zoomed in perspective we are standing too close to see the regularity of the engagement with affordances as it occurs in an entire sociomaterial practice (say the practice of architecture). Yet we do see another aspect of this landscape: we see how the details of the sociomaterial environment are changing and affordances are forming in the sociomaterial entanglement of people coordinating with others and materials in real-time. To make this concrete, let us turn to an example of sociomaterial coordination in action.

#### Sociomaterial Constitution in Practice

As an example of the sociomateriality of the landscape of affordances in flux, consider a situation in which one is having a coffee with a friend at a coffee bar. Coffee bars have become part of our human form of life; it is a behavior setting where the “recurrent features” of the coffee bar “both become[…] a resource for, and [are] organized by, customers speaking together” in coffee bars (Laurier, 2008, p. 168). Zooming in to the scale of the skilled individual entering into such a place for a drink, the way the room is furnished, the walls, the tables, the bar, the chairs, the people, turn out to entangle into a rich landscape of affordances in flux that enable and constrain the activities of an individual entering into the behavior setting: the welcoming smile of the waiter offers the affordance of ordering coffee, the friend affords having a conversation, the coffee cup affords grasping, the spoon stirring, the coffee drinking, the biscuit eating and the people to the right afford glancing at. Moreover, somewhere on the horizon of this situation, the 4PM train the person is planning to take back home will afford catching.

Looking at the sociomateriality in flux, we can see how social coordination and materiality are intermingling as affordances show up. As different affordances are coordinated with and responded to in appropriate ways, they change the sociomaterial environment – and thus the landscape of affordances shaping the unfolding situation. For example, during the conversation, the affordances of the words spoken by the friend and the affording coffee are coordinated with and get intertwined: “[T]he very fact of drinking … eases the conversation along. … Alongside this … the movements and objects that accompany drinking become resources in talking together” (Laurier, 2008, p. 178).

Consider how, at a later moment in the ethnographic transcript, a detail like placing a cup (in this case a glass) helps to shape the affordance to leave the coffee bar:

“After this quick sip F makes a charming and classic gesture of having finished with her drink even though the glass is not empty when she puts it back on the table: she pushes it away from her. The glass ending up slightly beyond the can of coke, a visible adjustment to the previous repeated return point of the glass to the table. By her pushing it away, she is establishing it, at this point in the unfolding action, as potentially the last sip from the glass.... F displays in this gesture, that she has noticed that B has finished her coffee and is now making available to B that they are potentially both finished with their drinks.” (Laurier, 2008, p. 175)

By gesturing (“social”) with the glass (“material”) and simultaneously changing the layout of the table (“material”) in subtle ways, a small part of the sociomaterial environment as a whole is reconfigured.^4^ Doing so, the local landscape of affordances changes for both people situated in it and the affordance to leave can become one *shared relevant possibility* among others.

Notice that from this perspective on a complex yet everyday situation in flux, as from the perspective on the form of life from afar, again everything is social and everything is material to some degree. Situated here in the landscape, the coffee spoon, the cup, the chairs, intermingle to become “resources in talking together” (Laurier, 2008, p. 178). Their materiality constrains the situation and helps to form a temporary “social synergy” (Marsh, 2015) that engages and constrains the behavior of both persons: “The unit they have formed will be resistant to forces that temporarily perturb the action” (Marsh, 2015, p. 23). Even the affordance to stand up and leave, which is coordinated to in the coffee bar situation by the two skilled talkers, is sociomaterial: both the flow of the conversation, the gesture and the change in table configuration enabled it. One would not manifest much skill in conversing if one were to stand up and leave the conversation halfway an unfinished sentence. The relevance of the possibility to stand up in this particular situation here and now, is neither just related to an embodied ability, nor is it just material or social – it is related to the constitutive entanglement of ability *and* sociomateriality.

#### Persistence through Change

This sociomateriality of affordances can be further highlighted when we imagine it is getting late and the 4PM train that one of the friends in the coffee bar needs to catch will leave very soon – she is in a hurry. When in a hurry, this concern of the individual will extend her situation, which means that it includes *coordinating to a larger part of the landscape* in which the individual is situated. Including the distant departure of the 4PM train within the situation will moreover re-configure many other sociomaterial aspects. For example, the frequency of the sips of coffee by the rushed person will increase and the topic discussed in conversation may be constrained. A moment of harmonious silence can now be the kind of opening in the conversation that moves the person to a slap on the thigh and the remark that it is time to leave (see Laurier, 2008). Although much in the behavior setting remains the same, in the newly unfolding sociomaterial context many affordances also change – even the temperature at which the coffee will afford drinking will be higher. In short, parts of the sociomaterial environment and the resulting behavior patterns are continuously re-arranged and reconfigured and other affordances enter the situation and dissipate as the departure time of the train approaches or the coffee is finished.^5^

From the perspective of a local observer that we adopted when zooming in on persisting practice in our form of life, we can thus see individuals in the process of coordinating skillfully. Any particular action within this process will be constrained by the available possibilities for acting in the form of life that we zoomed in on, and that the individuals that we see grew up in. As a particular action is unfolding, the particular sociomateriality of the *local* landscape of affordances will constrain the available actions further still – my pen with red ink will not afford drawing a blue line (see Rietveld and Kiverstein, 2014, p. 344) even though drawing blue lines is certainly a possibility available in our form of life.

Nonetheless, there is an important amount of uncertainty for the local observer, because the observed action can always continue in several directions – it has a kind of *indeterminacy* (Shotter, 1983; Schatzki, 2012, pp. 19–20) in the sense that what is done is yet to be determined – and can only be determined by the observer after the observed activity has been performed. In other words, namely viewed in terms of affordances: from the perspective of a local observer someone else’s particular unfolding action at a particular location in the landscape implies a multitude of possibilities which decrease in number as his or her action unfolds further until only one is realized (even though the situation will continue to afford many more actions).

To give an example of this increasing determinacy of action, consider that increasing the frequency of the sips of coffee allows not just for getting the 4PM train, but also for entirely different affordances such as making it to the bakery before it closes and for catching the 4PM movie. These possibilities are all available in the landscape of affordances in which the individual is situated. By continuously being *responsive* to (and constrained by) the relevant affordances available in the landscape however, the person turns left toward the station rather than right toward the bakery for example, and the possibility to go to the bakery moves further out of the individual’s situation (and other possibilities move in). All the while moreover the possibility to catch the 4PM train not only persists but also gains determinacy: the coordinated activity that started with increasing the frequency of sips at the coffee bar, ends in allowing little more than catching the 4PM train by jumping through the aperture of the closing doors of the train at the platform. At that point, the coordinated activity has realized the affordance to catch the 4PM train through coordinating sociomaterial aspects in a particular way, as was seen by the local observer, while of course many new possibilities for action have already entered the situation in which the individual is located.

Zooming in on the *nested actions* within the catching of the train, we see the same increasing determinacy of the coordinated activity even clearer. Again the space of possibilities available while acting will be constrained by the form of life, including the nesting affordance of catching the 4PM train and the behavior setting that the individual is a part of. The local observer might see for example that the person in the coffee bar is slightly moving forward toward the table. Limited by the narrow scope of the perspective of the local observer, it is uncertain what will happen next. Moving toward the table brings many affordances “within reach”: the possibility to stand up, to knock on the table, to indicate to a friend that it is time to leave, or to grasp the coffee cup. The action possibilities available in the local landscape are many. Again however, a person that lets herself be moved by the demands of the whole situation (including the 4PM train) responds in a way that the affordances attainable will, from the perspective of a local observer, decrease in number as her action unfolds until she in fact reaches and grasps the cup of coffee that she goes on to finish quickly. The indeterminacy of each act is continuously reduced during its unfolding until the relevant affordances are enacted in a certain unfolding sequence which reconfigures the particular sociomaterial entanglement.

What this zoomed in perspective shows is that the sociomateriality of the landscape of affordances that appears relatively *persistent* from a zoomed out perspective is *in flux* from the perspective of a local observer. When an individual acts he or she entangles sociomateriality and contributes to the regular ways of doing things available in the form of life. Which regularity of all the available regularities in the form of life the unfolding activity strengthens, however, is determined by what the individual does; in the unfolding sequence of the individual’s concrete activities. As the 4PM train is caught for example, it adds to our standing practice of catching trains, but not to that of going to the movie or to the bakery. In doing so, the skillful responsiveness of an individual’s situated activities contributed a tiny bit to keeping the affordance of taking trains available in our form of life – the individual *enacted* the affordance of catching trains (cf. Shotter, 1983). The landscape of affordances that is seen as *persisting* from a zoomed out perspective, turns out to be maintained, from the zoomed in observer’s perspective, by the multitude of ways in which ongoing coordination is entangled in sociomaterial situations in flux.

## Skilled Intentionality

In order to integrate social coordination and affordances into a common framework, the foregoing discussion of the SIF showed how social coordination and materiality are situated and entangled in the affordances available in the form of life, which we can see from a zoomed out perspective (the first perspective discussed). We moreover showed what such continuous intermingling looks like in terms of the ongoing coordination we find from a zoomed in (local observer’s) perspective on real-life situations (second perspective). However, we also aim to show how responding to affordances is always unfolding in concrete situations and how accounting for this in an integrative framework could extend the scope of ecological psychology. To show this we need to provide a third perspective on the form of life: we need the actor’s lived perspective which foregrounds the responsiveness to multiple relevant affordances of an individual that developed his or her skills within the form of life we are considering.

### Acting within a Field of Relevant Affordances

As mentioned above, Skilled Intentionality is defined as coordinating with multiple affordances simultaneously in a concrete situation (Rietveld et al., 2016). Individuals are enmeshed in a constellation of practices; in a form of life. In the SIF, acting individuals can be thought of as continuously forming aspects of the sociomaterial environment and thus as part of the landscape of affordances.

Skilled individuals are already entangled within the landscape of affordances (i.e., their partaking is implied by the “abilities” part of the definition of affordances as relations between aspects of the sociomaterial environment in flux and abilities available in a form of life). They can have *access* to a part of the landscape in so far as they have the skills to act on it (Noë, 2012).^6^ A skilled individual engages with, and continuously develops within a part of the landscape he or she cares about, which is lived as the “field of relevant affordances.”

The field of relevant affordances consists of the affordances that are currently significant to a skilled individual as he or she is engaging with a concrete situation. As mentioned above, it refers to the lived perspective, opened by the individual’s abilities and concerns, on a part of the landscape of affordances in flux. Experientially the field of affordances is made up by the relevant affordances that “stand out” among the rest of the landscape of affordances (De Haan et al., 2013; Bruineberg and Rietveld, 2014; Kiverstein, 2016). These attractive affordances are described as soliciting, or inviting, behavior (Dreyfus and Kelly, 2007; Withagen et al., 2012; Rietveld and Kiverstein, 2014). The soliciting character of these relevant affordances is the experiential equivalent of a bodily “action readiness” on the part of the skilled individual (Frijda, 1986, 2007). This preparation to act on relevant affordances is possible because of the abilities the individual has acquired thanks to a history of interactions in sociomaterial practices (Rietveld, 2008a).

These *relevant affordance-related states of action readiness* are crucial for understanding the interdependence of the skilled individual and his or her evolving situation as can be observed by a local observer or scientist (Bruineberg and Rietveld, 2014). Briefly, according to the SIF, a skilled individual has developed her abilities within the dynamics of the landscape of affordances of a form of life. The individual’s intrinsic dynamics can be understood as multiple bodily states of action readiness that are attuned to the *relevant* affordances in the situation. States of action readiness are reciprocally coupled to the landscape of affordances, in the sense that these states of action readiness self-organize and shape the selective openness to the landscape of affordances for the individual to accommodate the skilled individual’s concerns, i.e., to allow him or her to maintain or obtain sufficient grip on the situation. In this way, some affordances in the landscape show up as more and some as less relevant to the individual’s unfolding activities. The intrinsic dynamics of the individual’s states of action readiness thus allows for a selective openness to be responsive to the *relevant* affordances the individual encounters as it acts (for more on this see De Haan et al., 2013; Bruineberg and Rietveld, 2014; Kiverstein and Rietveld, 2015; Bruineberg et al., 2016).

### The Unfolding Action from the Actor’s Lived Perspective

The field of affordances brings us a final and crucial viewpoint on the form of life: it complements the first zoomed out, overlooking, perspective and the second zoomed in perspective of the local observer of someone’s activities with a *re-orientation* on the latter perspective, now from *within* the actions as they unfold. That is, thirdly, it adds to our perspectives on the form of life the means to understand the actor’s lived perspective – his or her *first person* experience. Viewed from within, the evolving landscape of affordances appears both as soliciting and as persisting. That is, while we lose track of some of the flux of patterned human activity over time, we gain a sense of skilled intentionality and a renewed view on the persistence of affordances.

Recall how the relative persistence of regularities in standing practices made way for continuous change of the sociomaterial environment as we zoomed in on the landscape of affordance. Now that we re-orient our perspective toward the actor’s lived perspective, these two phenomena – the persistence of the form of life that an individual grows up in, and the continuous change in the sociomaterial environment that acting implies – can be reconnected. To see how this would work we need to consider the continuity in the *history of the skilled individual* who acts.

From the lived perspective we experience the landscape of affordances in flux from within, on the basis of the continuity of our own history of skills as we have been growing up within our form of life (that we can see from a zoomed out perspective). In other words, our individual familiarity with our form of life is based on our history of skilled engagement^7^ (see also Heft, 1996; Rietveld, 2008a; Myin, 2016; Van Dijk and Withagen, 2016). From the lived perspective, the landscape of affordances in flux (that we could identify from a zoomed in observer’s perspective) shows up in terms of a multitude of possibilities for acting that are relevant to someone’s life and current concerns and solicit him or her to act on them. When acting to catch the 4PM train, the individual’s particular history within the form of life enables the person to be selectively responsive to those relevant affordances that move him/her toward the train station. In spite of the flux of the situation in which the skilled individual is engaged the person selectively responds to the affordances relevant to him/her.

With the actor’s lived perspective on the landscape of affordances in flux we thus connect with both the zoomed out perspective on the form of life and with the zoomed in perspective of the local observer. Notice that, as we have seen above, by selectively responding to the soliciting affordances in the individual’s field of relevant affordances, the skilled individual is in the process of contributing to the maintenance of these affordances as available in the form of life as a whole. Thus, the increasing determinacy of the act that we saw from the zoomed in perspective on the landscape of affordances, can return in the lived perspective. Here, however, it has a different character: we propose that a skilled individual can experience the increasing determinacy of action from *within* the unfolding act as “directedness” toward the relevant affordances available in the form of life that she is in the process of enacting. This unfolding enactment can be experienced pre-reflectively as having an “intentional” character (see cf. Shotter, 1983; Heft, 1989). Unlike the local observer, the acting individual herself will relatively seldom be uncertain about or surprised by the things that she does during the day, because action switches are often already announced by the pre-reflectively experienced attraction/allure of some of the relevant affordances in the field. Considering the skillful responsiveness to multiple nesting and nested affordances simultaneously, i.e., the responsiveness to a whole *field of relevant affordances*, an individual then manifests skilled intentionality in the context of his or her form of life (see Rietveld, 2008b; Bruineberg and Rietveld, 2014).

### Skilled Intentionality Unfolding in Architectural Practice

Finally, to show the merit of having these three complementary perspectives at our disposal within the Skilled Intentionality Framework, we turn to a case of skilled individuals who have learned to respond skillfully to the sociomaterial practices they are part of: we turn to architects working on a future building (a mobile sculpture). In doing so we aim to show how the sociomateriality of affordances can open up ecological theory to enable it to deal with what traditionally are considered “hard cases” such as dealing with non-existent things. Against the background of this example, in the discussion that follows we return to our starting point and consider the relation between social coordination and engagement with affordances by discussing how the SIF incorporates each and how it invites us to take a more situated approach in each case.

The nice thing about conceptualizing affordances as belonging to a form of life – i.e., in a fundamentally sociomaterial way – is that it can allow ecological psychology to move beyond the concrete-abstract distinction that is omnipresent in cognitive science. Consider a case where architects are designing a large mobile sculpture (taken from Rietveld and Brouwers, 2016). This sculpture, which is the size of a small house, is heavy and constructively requires such a large rear wheel that it might compromise the esthetics of the work of art. The architects start to determine how to proceed, using several affordances offered by the sociomaterial environment and creating some new ones:

“[Junior project leader AM] clicks on her computer, moving and changing lines, perspectives, colors, and scales; she makes adjustments and new sketches to then again revise these by adjusting lines and so on. … [S]he prints the five designs [and] walks over to [architect] RR, puts the printed drawings in front of him on the table when, while keeping their eyes focused on the prints, they pull up their chairs and stoop over the five designs. … RR picks up a pen, ticks off the second design, and then strikes it through: ‘This isn’t good.’ He then checks the fifth design: ‘Can the wheel rotate/turn around here?’ And what about the side-view/profile, what does it look like here?’ AM responds in a somewhat doubtful way, after which RR also strikes out this design. ‘Look, the wheel does not nicely connect here, in the other alternatives you have created more space at this point.”’ (Rietveld and Brouwers, 2016, p. 9)

Notice the many affordances that solicit and are acted on in a coordinated fashion: lines of the computer solicit changing, chairs afford pulling up and sitting next to each other, the pen solicits picking up and writing, a question affords answering, and the printed drawings solicit several comparisons. Coordinating with these nested affordances, just as in the 4PM train-example, entangles sociomaterial aspects as it *enacts* the nesting affordance of developing a good design.

Recall how the activity of catching the 4PM train was increasingly determined, and the affordance of taking trains enacted, in simultaneously coordinating to the standing practices in which trains run on time, the possibility of getting to the train station, of drinking coffee, and of paying the bill. Similarly here, in the process of realizing a satisfying design for the mobile sculpture, the design is increasingly determined by acting in accordance with the practices where the final design will have its place (e.g., as a mobile sculpture for public use and as part of an art collection) and simultaneously coordinating to the affordances offered by the printed drawings, the movable 3D-lines, the pen and a collaborator (Rietveld and Brouwers, 2016).

The right design therefore does not need to be “determined” in advance. There is no fully specified picture or description of the end result on the basis of which the design is realized. On the contrary, the design is realized in practice because it is getting increasingly determined or developed in acting within the landscape of affordances. The process of designing the sculpture can even have the determining, directed, character of nesting affordance for the architects, because they are in the process of enacting a satisfying design. For example, having the five different printed drawings affords a more precise conversation, within which they are evaluated one by one, compared and discarded in the process until finally one, it turns out, is selected for further development, which improves the architects’ grip on the final design and resolves a feeling of dissatisfaction or discontent (see Rietveld and Brouwers, 2016).

This kind of skilled intentionality is founded on a history of engaging in the relevant practices in which many details of the sociomaterial environment have been encountered (Rietveld, 2008a; Rietveld and Kiverstein, 2014; Myin, 2016). Having the ability to act in accordance with both the point and the details of the sociomaterial practice is having skill – in this case an architect’s skill. In the process of coordinating with an evolving field of relevant affordances offered by the sociomaterial environment, the architects tend toward grip on their design. Thus, although during this episode the mobile sculpture was still non-existent in a sense, it is perfectly concrete as the coordination with sociomaterial aspects of the environment is realizing the affordance of designing a mobile sculpture.

## Concluding Remarks

By making the human form of life the starting point for ecological psychology, and thus foregrounding sociomateriality in each situation, this paper showed how the Skilled Intentionality Framework integrates affordances with social coordination. In the SIF affordances are defined as relations between aspects of the sociomaterial environment in flux and abilities available in a form of life. By showing how the sociomaterial entanglement re-appears when taking three different perspectives on (i) the whole form of life – and the persistent landscape of affordances it implies, and (ii) the zoomed in perspective of an observing behavioral scientist or dynamicist observing an actor located at a particular place in the landscape of affordances and (iii) the lived perspective of person engaged in action – we showed what a situated, integrated take on engagement with affordances looks like. Thus we showed how different aspects of the notion of affordances and of coordination fit in: while theories and methods of (social) coordination tend to focus on the zoomed in observer’s perspective on the (inter)actions within an evolving landscape of affordances, those studying affordance perception are mostly focusing on affordances as we encounter them from our lived perspective – as agent-scaled perceived resources for action (e.g., Warren, 1984; Oudejans et al., 1996).

However, as we have stressed throughout this paper, these different viewpoints offer *complementary* (and not necessarily exhaustive) perspectives on the sociomaterial entanglement of the form of life as a whole (see Klaassen et al., 2010). The perspectives suggest that both fields of ecological psychology could consider broadening their scope in two principled ways. First, they could broaden the range of phenomena within their own perspective. As we have detailed, one is never coordinating with other people in isolation. The sociomateriality of the landscape of affordances in flux urges the study of social coordination to include coordination with materiality, i.e., as sociomaterial synergies. Moreover, zooming out emphasizes a focus not just at the scale of immediate interpersonal (e.g., dyadic) interaction, but to also include nesting scales of coordinating (Wijnants et al., 2012; Schmidt et al., 2014) with more distant dealings and places (Heft, 1996; Bruineberg and Rietveld, 2014; Van Dijk and Withagen, 2016) and perhaps even entire practices (Rietveld and Brouwers, 2016) and language games (Rietveld and Kiverstein, 2014; Van Dijk, 2016). Furthermore, from a lived perspective, one never encounters an affordance in isolation. Studies on affordances should thus take note of the sociomaterial context by studying affordance perception in the context of a field of relevant affordances embedded in a behavior setting (Heft, 2007, 2012) and/or a sociomaterial practice (Rietveld and Brouwers, 2016; Rietveld et al., 2016). By taking a more situated approach, both fields can thus contribute to the same overall goal of extending the reach of ecological psychology toward dealing with case of so-called “higher” cognition.

Second, as each of the three perspectives discussed foregrounds different aspects of the form of life, but backgrounds or neglects others, we believe each field should aim to keep an eye on at least one other perspective on the form of life to be able to claim to see the whole picture. Ethnography highlights the importance of this as it aims to link a zoomed in perspective on concrete situations, or interviews based on individual’s lived perspective, to the regularities at the level of the sociomaterial practice as a whole; i.e., to what we have called a zoomed out view on the form of life. Ecological psychology could thus benefit from including ethnographical methods and social sciences that thematize the patterned practice of the form of life (e.g., Roepstorff, 2008). That way we can get a clear view of the richness of the landscape of available affordances offered by our evolving sociomaterial environment (e.g., Malafouris, 2014) as it persists and changes within cultures, communities, and behavior settings.

The view that we have presented in its multitude of perspectives on the whole does not need to rely on (ontological) priorities, in the sense that it does not need to presuppose a hierarchical and pre-structured world (see Van Dijk and Withagen, 2014; Van Dijk, 2016; see also Hodges and Baron, 1992; Ingold, 2011). For example, the notion of “higher” cognition that we discussed is indicative of a supposed hierarchy, but its use can be avoided once we realize that the phenomenon the notion aims to single out (e.g., architects designing a novel sculpture) amounts to adequately coordinating with multiple affordances simultaneously across increasing scales of sociomateriality. Conceptually this required the notion of a constitutive entanglement. One of the merits of our view on the constitutive entanglement is that it ties our concepts in with a *process* that constitutes the whole while forming its parts. In this way it opens up our theory to the scrutiny of dynamical methods, in which it is common to distinguish between macro-level patterns of activity and micro-level patterns of activity. For example, we can formalize the dynamics of the agent-environment system as a whole, or focus on a part and use the tools and concepts of dynamical systems theory to increase our understanding of the dynamics of multiple simultaneous affordance-related states of action readiness (see Bruineberg and Rietveld, 2014; Bruineberg et al., 2016).

Finally, in a fragment quoted by Costall (1997), Gibson tried to clarify how affordances are objective and subjective through their relational persistence: “Affordances are both objective and persisting and, at the same time, subjective, because they relate to the species or individual for whom something is afforded” (Gibson, 1982, p. 237). Our view makes sense of this idea by showing how the distinction between the individual and the “species” is not the most relevant one. By rather talking about a *form of life* by focusing more on “*how* an animal lives than [on] *where* it lives” (Gibson, 1979, p. 128), i.e., on its way of life, we showed that affordances are both persisting environmental resources which can solicit an individual and persisting relations in the ecological niche. Yet they are continuously forming in the multitude of our activities that make up our form of life.

## Author Contributions

LvD and ER: conception of the work, drafting the work.

## Conflict of Interest Statement

The authors declare that the research was conducted in the absence of any commercial or financial relationships that could be construed as a potential conflict of interest.

## Footnotes

1 There is in important normative aspect to this kind of skilled engagement in a form of life or socio-cultural practice. Such “situated normativity” can be seen as distinguishing between better and worse (e.g., adequate and inadequate, appropriate and inappropriate, or correct and incorrect) in the context of a concrete situation (Rietveld, 2008a). We have analyzed this kind of situated normativity in earlier work, both for unreflective skilled action (Rietveld, 2008a, 2010) and for more reflective forms of skilled action, such as seeking the right word (Klaassen et al., 2010), evaluating the quality of an architectural design (Rietveld and Brouwers, 2016) or making a correct explicit judgment about something (Rietveld and Kiverstein, 2014; Kiverstein and Rietveld, 2015).

2 The interdependence is also highlighted in the way Gibson (1979) conceives of the relation between an ecological niche, i.e., a “set of affordances” (Gibson, 1979, p. 128) that implies a way of life, and a kind of animal: “The natural environment offers many ways of life, and different animals have different ways of life. The niche implies a kind of animal, and the animal implies a kind of niche. Note the complementarity of the two.” (Gibson, 1979, p. 128).

3 Note that in this view, as a process the whole is itself never complete. It is fundamentally open to change.

4 Note that a closer examination of the gesturing, glass and table layout would reveal that all three are sociomaterial themselves rather than either social or material.

5 What this approach does is taking seriously the flux of the sociomaterial environment (see Ingold, 2000, 2011 for the importance of this). This is crucial for dealing with many real-life situation. Think for instance of situations of crossing a busy street by foot. The affordances for crossing the street open up and dissipate (discontinuously) all the time.

6 Note that certain types of power and exclusion in society can make it the case that someone with the right skills still does not succeed in getting access to an affordance. A discussion of this political dimension of affordances in the context of the SIF will have to wait to another occasion.

7 Below we will see that abilities are acquired in concrete situations in sociomaterial practice. This process of enskilment is typically scaffolded by more experienced practitioners in a process that can be characterized as education of attention (see Rietveld, 2008a; Rietveld and Kiverstein, 2014).
